# Inkoo and Sindbis viruses in blood sucking insects, and a serological study for Inkoo virus in semi-domesticated Eurasian tundra reindeer in Norway

**DOI:** 10.1186/s12985-022-01815-0

**Published:** 2022-06-03

**Authors:** Ruchika Shakya, Morten Tryland, Rose Vikse, Javier Sánchez Romano, Kjetil Åsbakk, Ingebjørg H. Nymo, Reidar Mehl, Magnus Evander, Clas Ahlm, Olli Vapalahti, Olivia Wesula Lwande, Niina Putkuri, Wenche Johansen, Arnulf Soleng, Kristin S. Edgar, Åshild K. Andreassen

**Affiliations:** 1 Present Address: Virology Unit, Faculty of Veterinary Medicine, Norwegian University of Life Sciences, Ås, Norway; 2 Department of Arctic and Marine Biology, UiT The Arctic University of Norway, Tromsø, Norway; 3 Division for Infection Control and Environmental Health, Department of Virology, Norwegian Institute of Public Health, Oslo, Norway; 4 Present Address: Department of Medical Biology, UiT The Arctic University of Norway, Tromsø, Norway; 5 Present Address: Section for Food Safety and Animal Health, The Norwegian Veterinary Institute, Tromsø, Norway; 6 Section of Pest Control, Norwegian Institute of Public Health, Oslo, Norway; 7 Department of Clinical Microbiology, Umeå University, Umeå, Sweden; 8 Department of Virology, University of Helsinki, Helsinki, Finland; 9 Department of Veterinary Biosciences, University of Helsinki, Helsinki, Finland; 10 Finnish Red Cross Blood Service, Helsinki, Finland; 11 Department of Biotechnology, Faculty of Applied Ecology, Agricultural Sciences and Biotechnology, Inland Norway University of Applied Sciences, Hamar, Norway; 12 Department of Natural Sciences and Environmental Health, Faculty of Technology, Natural Sciences and Maritime Sciences, University of South-Eastern Norway, Bø, Norway; 13 Department of Forestry and Wildlife Management, Faculty of Applied Ecology, Agricultural Sciences and Biotechnology, Inland Norway University of Applied Sciences, Evenstad, Norway

**Keywords:** Arbovirus, Blood sucking insects (BSI), Mosquito, Reindeer, INKV, SINV, Prevalence, Estimated pooled prevalence, IIFA, Seroprevalence

## Abstract

**Background:**

Mosquito-borne viruses pose a serious threat to humans worldwide. There has been an upsurge in the number of mosquito-borne viruses in Europe, mostly belonging to the families *Togaviridae,* genus *Alphavirus* (Sindbis, Chikungunya), *Flaviviridae* (West Nile, Usutu, Dengue), and *Peribunyaviridae,* genus *Orthobunyavirus,* California serogroup (Inkoo, Batai, Tahyna). The principal focus of this study was Inkoo (INKV) and Sindbis (SINV) virus circulating in Norway, Sweden, Finland, and some parts of Russia. These viruses are associated with morbidity in humans. However, there is a knowledge gap regarding reservoirs and transmission. Therefore, we aimed to determine the prevalence of INKV and SINV in blood sucking insects and seroprevalence for INKV in semi-domesticated Eurasian tundra reindeer (*Rangifer tarandus tarandus*) in Norway.

**Materials and methods:**

In total, 213 pools containing about 25 blood sucking insects (BSI) each and 480 reindeer sera were collected in eight Norwegian reindeer summer pasture districts during 2013–2015. The pools were analysed by RT-PCR to detect INKV and by RT-real-time PCR for SINV. Reindeer sera were analysed for INKV-specific IgG by an Indirect Immunofluorescence Assay (n = 480, IIFA) and a Plaque Reduction Neutralization Test (n = 60, PRNT).

**Results:**

*Aedes* spp. were the most dominant species among the collected BSI. Two of the pools were positive for INKV-RNA by RT-PCR and were confirmed by pyrosequencing. The overall estimated pool prevalence (EPP) of INKV in Norway was 0.04%. None of the analysed pools were positive for SINV. Overall IgG seroprevalence in reindeer was 62% positive for INKV by IIFA. Of the 60 reindeer sera- analysed by PRNT for INKV, 80% were confirmed positive, and there was no cross-reactivity with the closely related Tahyna virus (TAHV) and Snowshoe hare virus (SSHV).

**Conclusion:**

The occurrence and prevalence of INKV in BSI and the high seroprevalence against the virus among semi-domesticated reindeer in Norway indicate that further studies are required for monitoring this virus. SINV was not detected in the BSI in this study, however, human cases of SINV infection are yearly reported from other regions such as Rjukan in south-central Norway. It is therefore essential to monitor both viruses in the human population. Our findings are important to raise awareness regarding the geographical distribution of these mosquito-borne viruses in Northern Europe.

**Supplementary Information:**

The online version contains supplementary material available at 10.1186/s12985-022-01815-0.

## Introduction

Mosquito-borne viruses emerge as a group that poses a serious threat to human health worldwide [1]. There has been an upsurge in the number of mosquito-borne viruses in Europe mostly belonging to the families *Togaviridae* (Sindbis virus SINV, Chikungunya virus), *Flaviviridae* (West Nile virus, Dengue virus), and *Peribunyaviridae* (Inkoo virus INKV, Batai virus BATV, Tahyna virus TAHV) [2, 3].

Surveillance of mosquito-borne viruses and their prevalence in the human and animal populations is highly required, since INKV, SINV, Chikungunya virus and West Nile virus infections often are not reported and remain undiagnosed [2]. Among other mosquito-borne viruses, INKV and SINV are known to be circulating in Norway, Sweden [1, 4, 5], Finland, and some parts of Russia [6–9]. In humans, INKV causes mild fever to fatal encephalitis while SINV causes arthritis and rashes [10, 11].

INKV, a member of the California serogroup together with TAHV and Snowshoe hare virus (SSHV), is an enveloped virus belonging to the genus *Orthobunyavirus* in the *Peribunyaviridae* family with a tri-segmented, negative-sense, single-stranded (ss) 12.4-kb (kilo-base pair) RNA genome [12]. Phylogenetically, INKV is closely related to Jamestown Canyon Virus (JCV) found in the US [13, 14]. INKV has been detected previously in adult *Aedes (Ae.) communis*, *Ae. hexodontus* and *Ae. punctor* mosquitoes, and in *Ae. communis* larvae, although there is a necessity for identification of mosquito vectors capable of transmitting INKV [7, 15–17]. INKV has been found to be circulating in Northern Europe, including Norway [2, 16]. Cattle (*Bos taurus*), reindeer (*Rangifer tarandus*), moose (*Alces alces*), red fox *(Vulpes vulpes)* and snow hare (*Lepus timidus*), are considered to be major vertebrate hosts of INKV besides humans [6].

SINV is a member of the Western equine encephalomyelitis virus complex. It is an enveloped, positive sense, 11.7-kb ssRNA virus belonging to the genus *Alphavirus* in the *Togaviridae* family. Wild birds are considered as virus reservoir [18]. Antibodies against SINV have been detected in migratory and residential birds [19, 20]. Ornithophilic mosquitoes (mosquitoes that feed on birds) of the genera *Culex* (*Cx.*), and *Culiseta* as well as *Ochlerotatus* spp*.*, and *Aedes* spp*.* are considered to be the major SINV vectors [2]. Experimental infection studies implicate *Cx. torrentium* as a competent vector for SINV in northern Sweden [21–23]. The vector species of SINV in Sweden prefer lowland forested wetlands and humid forests with deciduous and coniferous trees as habitats [19]. SINV causes human arthritic diseases [24, 25], known by various names in Fennoscandia; “Bærplukkersyken” in Norway [26], Ockelbo disease in Sweden [27], Karelian fever in Russia [28], and Pogosta disease in Finland [6].

Both INKV and SINV have been isolated from diverse species of mosquitoes including *Ae. cinereus*, *Cx. torrentium*, *Cx. pipiens, Culiseta morsitans, Ae. Communis,* and *Ae. punctor* [8, 29]. Moreover, evidence pointing to vertical transmission of these viruses in vectors has been demonstrated through detection of viral RNA in mosquito larvae [1]. In addition, SINV has been suggested to overwinter in *Cx. pipiens* mosquitoes [30]. Antibodies against INKV and SINV have been detected in human populations in Sweden and Finland [31–33]. In Russia, chronic neurological disease was reported in patients with antibodies to INKV [34]. Acute human INKV infections in association with encephalitis have been reported in Finland [11].

In Norway, INKV and SINV have not been studied extensively despite findings of viruses in humans and mosquitoes in 1978 and 1992 [7, 35, 36]. There is a lack of knowledge covering the distribution and prevalence of these viruses in BSI vectors, humans, potential reservoir species, and the regions affected. Other California Encephalitis (CE) group viruses now categorized as California serogroup viruses have previously been isolated in Norway, but the methods used to identify them were not specific enough to confirm if it was INKV or related viruses [16]. There are several cases of encephalitis reported to Norwegian Surveillance System for Communicable Diseases (MSIS) with unknown aetiology [26]. These may be associated with INKV or other vector-borne pathogens. SINV cases are not reported to the MSIS but about 15–20 clinical cases have been diagnosed since 1982.

A recent study indicated the need of monitoring INKV in vertebrate hosts such as moose and reindeer to identify the possible risk of transmission to humans [29]. The present study aimed to provide new knowledge on the prevalence and distribution of INKV and SINV in blood sucking insects, and to investigate their exposure in semi-domesticated reindeer in Norway. The combined analysis of blood sucking insects and reindeer serum samples collected on the reindeer summer pastures provides an indication of the distribution of these viruses in Norway, which could be addressed further and evaluated as potential causes of human encephalitis in Norway.

## Materials and methods

### Study area and collection of samples

#### Blood sucking insects

**Fig. 1:**
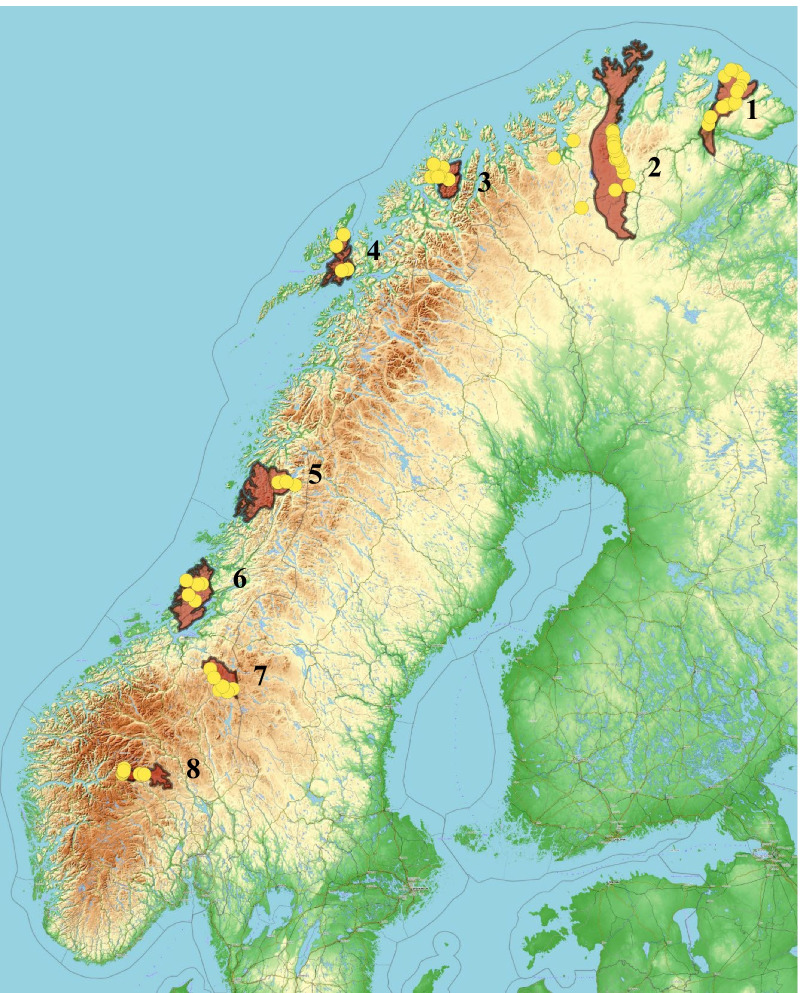
The map illustrates the sampling locations where the blood sucking insects (BSI) and reindeer sera were collected. 1: Tana, 2: Lakselv, 3: Tromsø, 4: Lødingen, 5: Hattfjelldal, 6: Fosen, 7: Røros, and 8: Valdres. The brown areas represent the summer pastures for each of the reindeer herds, whereas the yellow dots indicate the sampling locations for blood sucking insects (BSI) in or in the vicinity of each summer pasture

Mosquitoes and midges, hereafter named blood sucking insects (BSI), were collected by The Arctic University of Norway (UiT) as a part of the research project Climate and Reindeer Diseases (CARD), during the summer season between July and August each year from 2013 to 2015. BSI were caught with a mosquito trap (Mosquito Magnet Independence; Woodstream® Corporation, Pennsylvania, USA), using propane gas to produce carbon dioxide (CO_2_) and a biting insect attractant (Mosquito Magnet Octenol, Woodstream® Corporation). The trap was set to run for two to 24 h depending on the abundance of BSI and weather conditions. The mosquito trap attracts all kinds of BSI and were collected for several research purpose. To cover a variety of different BSI species the sampling was performed at four to 24 multiple sites from each of the eight reindeer summer pasture locations and whenever possible, each site was visited twice during the summer season (Table 1, Fig. 1). One of the main purposes of sampling BSI for this study was to test if there were any mosquito-borne viruses, like INKV and SINV, within these pools. BSI were killed by freezing and stored at − 20 °C from 2 days and up to 2 weeks after capture, until a − 80 °C freezer was available. The BSI were collected in cryotubes (1.8 ml, Thermo Fisher Scientific, Rochester, NY, USA) with appr. 25 insects per tube from each site. One pool from each site were analyzed for detection of INKV and SINV.

**Table 1:** An overview of selected summer pastures for semi-domesticated reindeer (*Rangifer tarandus tarandus*)

County	Locations	UTM-coordinates*	
		Latitude (S–N)	Longitude (E–W)
Troms and Finnmark	Tana	35 W 0573349	7820819
Troms and Finnmark	Lakselv	35 W 0441724	707695
Troms and Finnmark	Tromsø	34 W 0429573	7738767
Nordland	Lødingen	33 W 0540824	7589369
Nordland	Hattfjelldal	33 W 0458873	7281479
Trøndelag	Fosen	32 W 0568340	7129347
Trøndelag	Røros	32 V 0619596	6966550
Innlandet	Valdres	32 V 0490781	6794290

Serum samples were collected during the following winter seasons (November–April) of 2013–2014, 2014–2015, and 2015–2016. Blood sucking insects (BSI) were collected from the reindeer summer pastures during the summer seasons (July–August) each year from 2013–2014, 2014–2015, and 2015–2016

^*^UTM-coordinates Universal Transverse Mercator coordinates

#### Reindeer serum samples

Blood samples were collected from 480 semi-domesticated reindeer grazing on eight different summer pastures (Table 1, Fig. 1). Reindeer were sampled about 3–6 months after the BSI sampling, i.e., during the winter seasons (November–April) of 2013–2014, 2014–2015 and 2015–2016, with the exception of the Lødingen herd which was not sampled during the winter 2015–2016. Adult reindeer males were underrepresented compared to adult females due to restricted availability in the herd at the time of sampling. From live animals, blood was obtained by venepuncture of external jugular vein using a venoject needle (Terumo, Leuven, Belgium) and blood collecting tubes (BD Vacutainer®; BD, Plymouth, UK). From slaughtered animals, blood was collected directly in the blood collecting tubes when bled. Blood tubes were centrifuged at 3500 rpm for 10 min to collect the serum. Sera were stored at − 20 °C until analysis [37].

#### Extraction of total nucleic acid from BSI

Each of the 213 pools of BSI were transferred to 2 ml tubes containing six steel beads (MP Biomedical Life Science, CA, USA) and 350 μl of RLT® lysis buffer with β-mercaptoethanol in a ratio of 1:10 (RNeasy Mini Kit, QIAGEN Inc., Valencia, CA, USA). They were homogenized at 4 °C by FastPrep®-24 5G instrument (MP Biomedical Life Science, CA, USA) operated at a relative velocity of 4.0 m/s for 60 s. The homogenates were centrifuged at 20,817×*g* for 5 min to remove BSI debris. The total nucleic acid (TNA) was extracted with MagNA Pure LC 2.0 (software v 1.1.24) instrument and MagNA Pure LC Total Nucleic Acid High-Performance Isolation kit (Roche Diagnostics GmbH, Penzburg, Germany) according to the manufacturer’s protocol. The elution volume of TNA was 60 μl.

#### Detection of INKV by one-step RT-PCR

To detect INKV, a RT-PCR amplifying the fragment targeting the gene encoding the non-structural protein (nsp1) of the Lövånger strain of INKV (GenBank accession number KX554935) was carried out. TNA extracted from BSI were amplified by RT-PCR in a 2720 thermal cycler (Applied Biosystems, Foster City, CA, USA) using SuperScript™ III One-Step RT-PCR System with Platinum™ Taq DNA polymerase kit (Invitrogen, Carlsbad, CA, USA). The amplification was carried out in a total volume of 25 µl per reaction, containing 20 µl mastermix and 5 µl extracted TNA template. The concentration of each primer and cycling conditions were modified from [1, 13, 38]. The concentrations of the INKV forward and reverse primers were 0.25 μM each. The optimized cycling conditions were: 55 °C for 30 min, 95 °C for 2 min followed by 45 cycles of 95 °C for 15 s, 60.5 °C for 30 s and 72 °C for 1 min, before a final extension at 72 °C for 5 min and 4 °C until the PCR products were further analysed. The PCR products were visualized on E-Gel® 48 Gels (2% agarose) with EtBr according to the manufacturer’s protocol (Invitrogen, Carlsbad, CA, USA) and stored at − 80 °C until further analysis. A tenfold serial endpoint dilution (10^–1^ to 10^–4^) of INKV positive controls and RNase free water as negative control were included in each test.

#### Detection of SINV by one-step RT-real-time PCR

Total nucleic acid from pooled BSI were analysed to detect SINV RNA by probe-based RT-real-time PCR, using qScript™ XLT One-step RT-real-time PCR ToughMix® kit (Quanta Biosciences, Gaithersburg, MD, USA) in a Rotor-gene 6000 (QIAGEN, Hilden, Germany). The primers and probe used for the amplification targeted the non-structural gene (nsp1) from the Ockelbo strain of SINV (GenBank accession number M69205.1) [1, 39]. The optimized cycling conditions for the reaction were: 55 °C for 30 min, 95 °C for 2 min followed by 45 cycles of 95 °C for 15 s, 60 °C for 30 s and 72 °C for 1 min, before a final extension at 72 °C for 5 min. The PCR products were stored at − 80 °C until further analysis.

Included on each plate was SINV RNA positive control, from the Lövånger strain, cultured in the lab, in a tenfold serial endpoint dilution (10^–4^ to 10^–7^) along with two RNase free water samples as negative control.

#### Pyrosequencing

All the PCR positive samples for INKV RNA were further analysed by Pyrosequencing [40], for sequence analysis (SQA) with the BioTage (Pyromark Q24) system (QIAGEN, Hilden, Germany). A tenfold serial endpoint dilution (10^–1^ to 10^–4^) of INKV positive controls and RNase free water as negative control were included in each test.

The positive controls were used as a standard to compare the sequences obtained from pyrosequencing of PCR positive samples. Samples with sequence similarity above 70% was regarded as positive in this study. Sequences were then aligned to previously identified INKV positive control and GenBank sequences using the Basic Local Alignment Search Tool (BLAST) provided by National Centre for Biotechnology Information.

#### Barcoding/speciation of BSI

Barcoding/speciation of BSI (10% of the total pool) was conducted on the same sample tube/pool that was subjected to the RT-PCR and pyrosequencing.

Amplification of the 710-bp fragment of the mitochondrial cytochrome c oxidase subunit I gene (COI) was performed. Briefly, amplification of cDNA from 10% of the total number of pools was performed using Phusion Green Hot Start II High-Fidelity PCR Master Mix (Thermo Fisher Scientific) with universal DNA primers namely; LCO1490 and HCO2198 as described by [41]. For each reaction, 2 μl of template was used together with 10 μl of 2 × Phusion mix, 1.25 μl of both forward and reverse primers (10 pmol), 0.6 μl of DMSO and 4.9 μl of nuclease free water, up to a total reaction volume of 20 μl. Conditions for reactions were 98 °C for 30 s for initial denaturation. Further, amplification was performed using 35 cycles of: 98 °C for 7 s, 50 °C for 15 s and 72 °C for 20 s. Final extension was performed at 72 °C for 7 min. PCR product was analysed by gel electrophoresis using 1.2% agarose in 1 × TAE with GelRed (Biotium Inc. Hayward, CA, US) and later purified with ExoSAP-IT kit (Thermo Fisher Scientific) and sent to Eurofin Genomics (Germany) for Sanger sequencing. Sequences were then aligned to previously identified mosquito species in GenBank using the Basic Local Alignment Search Tool (BLAST) provided by National Centre for Biotechnology Information.

#### Calculation of prevalence

Two hundred and thirteen pools with approximately 25 BSI in each pool were analysed. The sample size was designed for other research purposes by the CARD project. Estimated pooled prevalence (EPP) of INKV was calculated by the Epitools Epidemiological calculators [42]. The estimated prevalence was assumed to be close to zero with 95% confidence interval (CI) using Method 2 [43]. This method utilizes the frequentists approach assuming a fixed pool size, and perfect (100%) test sensitivity and specificity for the estimation of prevalence and confidence limits [40].

### Serological screening of reindeer samples

#### Indirect immunofluorescence assay (IIFA) for detection of IgG antibody against INKV

Slides were fixed with INKV antigen, a positive fluorescence signal from 40–50% of the cells was required for a positive result. The samples evaluated as borderline had a positive fluorescence signal from less than 40% of the cells [33]. Two INKV positive controls; anti-Tahyna mouse-ascites and INKV positive deer whole blood were included in each run.

Initially, mouse ascites (1:100), deer whole blood, and reindeer serum samples (1:20), were diluted in 1X PBS (Dulbecco’s solution A, pH = 7.4, Norwegian Institute of Public Health, Oslo, Norway).

Twenty μl of the diluted reindeer sera (n = 480) and the controls were analysed on slides and incubated at 37 °C for 1 h, and washed with cold PBS and water. The slides were stained with anti-mouse Alexa Fluor® 488 conjugate (Invitrogen Life Technology, Inc., Carlsbad, CA, USA) (dilution1:30) and anti-deer IgG fluorescein isothiocyanate conjugate (FITC) (KPL, Gaithersburg, MD, USA) (dilution 1:25) for 30 min at 37 °C and washed as described previously. Each slide was air dried, coated with glycerol before examining with a fluorescence microscope (Nikon Eclipse Ci, Nikon Corporation, Tokyo, Japan). The overall seroprevalence of IgG against INKV in reindeer sera was calculated as the percentage of the total number of positives of the total number of samples tested.

#### Cytopathic effect neutralization test (CPE-NT) from reindeer sera

Fifty-five reindeer sera samples evaluated as borderline from the IIFA analysis were further verified by the CPE-neutralization test at the Department of Virology, University of Helsinki as previously described [33].

#### Plaque reduction neutralization test (PRNT)

Reindeer sera (n = 60) with strongly positive (25), borderline (25), or negative (10) result by IIFA were confirmed with a PRNT to determine whether the IgG antibodies were specific for INKV, TAHV, or SSHV as previously described [32]. A 50% plaque reduction neutralization titer (PRNT_50_) was calculated as the reciprocal of the highest serum dilution based on 50% or greater reduction in the plaque counts. Samples with titre < 20 was considered negative and titres ≥ 20 were defined as INKV seropositive.

#### Statistical method for calculation of seroprevalence

All statistical analyses were performed by the program STATA Software v. 16 (Stata Corporation, College Station, TX, USA). The statistical difference between infection rate within each location were analysed by *χ*^2^ for each year. In addition, the interaction between infection, location, and year was analysed by logistic regression. In all analyses statistical significance was indicated by *p* < 0.05.

## Result

### Identification of blood sucking insects

Two-hundred and thirteen pools of BSI were visually identified with regards to species [44]. Besides *Culicidae* (mosquitoes), the BSI samples also contained *Simuliidae* (black flies), *Culicoides* (biting midges). *Aedes* spp. was the most common identified mosquito species with very few numbers of *Culex* spp. Of the mosquitoes identified were *Ae. excrucians*, *Ae. punctor/hexodontus*, *Ae. communis*, *Ae. nigripes, Ae. impiger, Ae. cinereus, Ae. diantaeus, Ae. pionips*, and *Culiseta* spp. that were most frequently observed from all different locations during the sampling period. *Ae. punctor* and/or *hexodontus, Ae. diantaeus* and/or *Ae. impiger* were the dominating species of *Aedes* mosquitoes in the BSI pools from the two INKV positive locations, Fosen and Røros. Similarly, *Culiseta* spp. could be identified from one sample from each of the two locations. *Ae. excrucians* and *Ae. cinereus* were only recorded in Røros.

In order to confirm the results from the visual identification barcoding analysis was performed. These results from randomly selected BSI samples (10%) indicated most of the identified pools as arthropods in addition to mosquitoes. Many of the pools contained mosquito species but other contained midges. The barcoding method only amplifies the most common, or best fit, not all insect species in the pools. Eight pools of BSI from Tromsø indicated that four of the pools contained a majority of midges *Prosimulium hirtipes*, two contained a majority of mosquitoes, *Ae. communis*, while the remaining two had a majority of fungus gnats *Marcocera spp.* and biting midges *Culicoides chioptera*. Similarly, eight pools from Lødingen demonstrated six pools of mosquitoes, four with *Ae. punctor* and/or *Ae. hexodontus*, two of *Ae. communis*, and two containing a majority of midges, *P. hirtipes*. One pool each from Fosen and Røros indicated a majority of the two midge's species, *Simulium monticola* and *P. hirtipes,* respectively.

### Screening for INKV by one-step RT-PCR

Two out of 213 analysed pools of 25 BSI were positive for INKV by RT-PCR. The results from these two positive pools were further verified as INKV by pyrosequencing (Table 2). One of the positive pools had 88% and the other had 94% sequence similarity to the INKV positive controls (10^–1^ to 10^–4^). The two positive pools were from Fosen and Røros from 2014. From this, the overall EPP was calculated to be 0.04% (95% CI) by the Epitools Ausvet calculator [42], with a site EPP of 0.5%.

**Table 2:** Analysis of blood sucking insects (BSI) for detection of INKV from eight different reindeer pastures in Norway by RT-PCR and pyrosequencing

Locations	Number of pools of BSI analysed			Number of pools positive for INKV by RT-PCR	Number of pools positive by pyrosequencing
	2013–2014	2014–2015	2015–2016		
Tana	5	9	8	0	0
Lakselv	12	26	18	0	0
Tromsø	1	8	7	0	0
Lødingen	8	8	8	0	0
Hattfjelldal	8	8	8	0	0
Fosen	8	8	8	1*	**1**
Røros	8	8	8	1*	**1**
Valdres	8	8	7	0	0
Total = 213				2	2

Bold indicates the positive samples and they were collected in 2014

* = the two positive pools were collected in 2014

### Screening for SINV by one-step RT-real-time PCR

None of the analysed pools of BSI were positive for SINV on RT-real-time PCR.

### IgG against INKV in reindeer sera by indirect immunofluorescence assay

Sera from 480 reindeer, representing eight different reindeer pastures in Norway were analysed for detection of IgG antibodies against INKV by indirect immunofluorescence assay (IIFA). In total, 296 (62%) serum samples tested positive with INKV-IIFA.

**Fig. 2:**
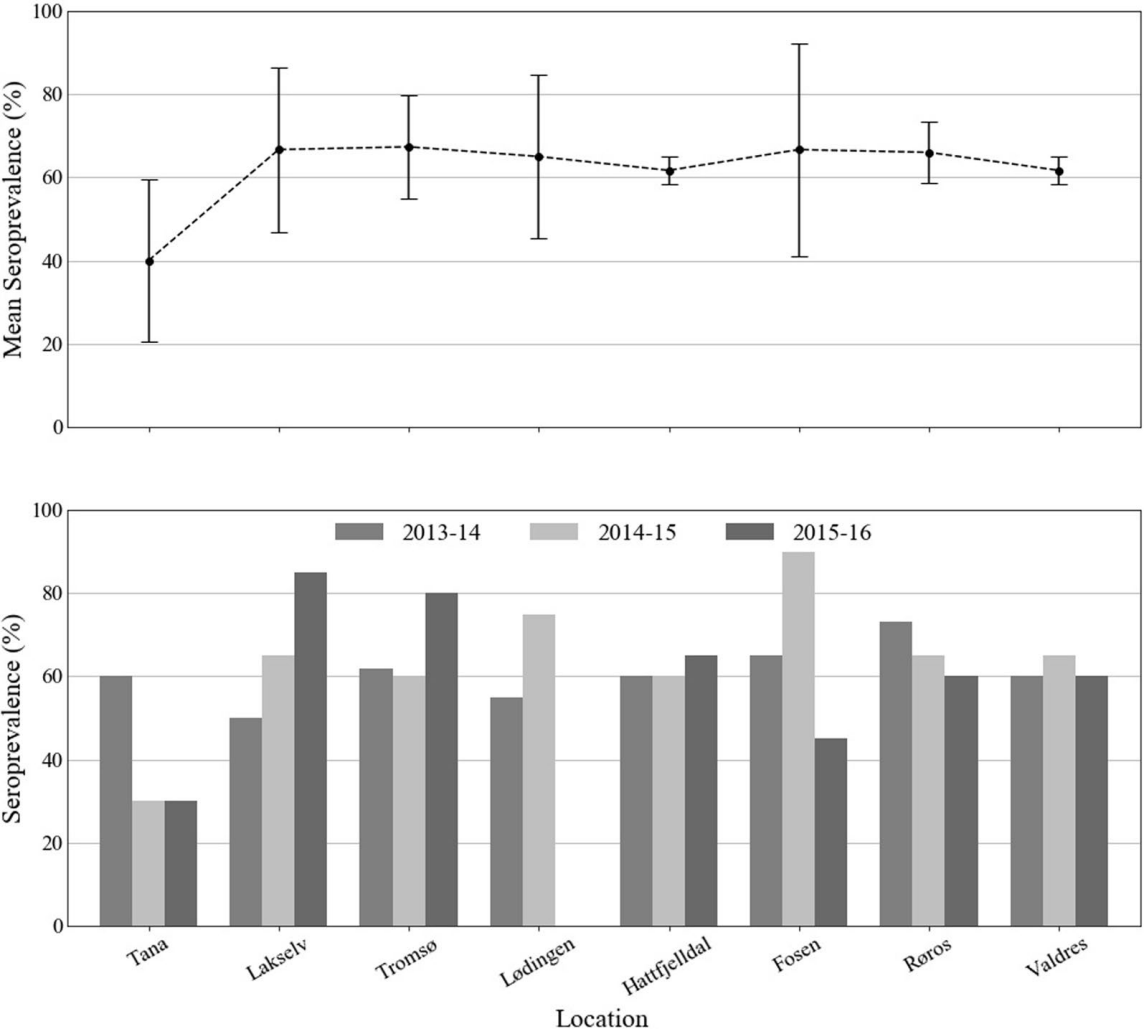
Seroprevalence (%) of INKV (IgG) in reindeer sera determined by IIFA from Tana, Lakselv, Tromsø, Lødingen, Hattfjelldal, Fosen, Røros, and Valdres from the winter seasons of 2013–2014, 2014–2015 and 2015–2016, indicated by bar graphs (bottom). Above each bar graph, the dots indicate the mean seroprevalence and the whiskers represent the confidence interval (95% CI) of each site throughout the year (2013–2014, 2014–2015, 2015–2016) (top)

IgG antibodies against INKV were detected in reindeer from all the eight different reindeer pastures of Norway (Fig. 2, Additional file 1: Table S1).

The IIFA results illustrated a high average seroprevalence of 67% in Lakselv, Tromsø, and Fosen while the lowest average seroprevalence of 40% was in Tana. There was no statistically significant difference in seroprevalence among the eight different reindeer pastures for the season 2013–2014 (50–73%). However, there was a significant difference between the locations both in 2014–2015 (*p* = 0.011) and 2015–2016 (*p* = 0.006) (*χ*^2^ test, between eight locations, STATA). Fosen 2014–2015 had the highest seroprevalence reaching up to 90% with an OR of 6.0 (95% CI [1.1–33.3]) *p* > 0.04, while Tana (2014–2015) had the lowest seroprevalence of 30% with an OR of 0.2 (95% CI [0.1–0.9]) *p* > 0.03 (logistic regression analysis, STATA). Similarly, for the winter season 2015–2016, 30% of the reindeer samples from Tana were seropositive and the highest seroprevalence was detected in Lakselv (85%).

Of the seropositives, 37% (175/480) of the reindeer were female, 23% (112/480) were male, and 2% (9/480) of unknown gender (Table 3). The seroprevalence of INKV among the calves (< 1 year of age) indicate recent exposure to the virus (previous summer). The highest number of seropositive were among adult females, 36% (107/296) (Table 3).

**Table 3:** Seropositivity (%) of IgG antibodies against INKV reindeer sera determined by IIFA for the winter seasons of 2013–2014, 2014–2015, and 2015–2016, based on age (Adult =  > 1 year, Calves =  < 1 year) and sex presented as seropositive/tested (%)

	Seropositive (n)*	% of total (480)	% of total seropositive (296)
Male adults	28	6	9
Male calves	84	18	28
Female adults	107	22	36
Female calves	68	14	23
Unknown adults	0	0	0
Unknown calves	9	2	3
Total	296	62	100

^*^n = Number

### Cytopathic effect neutralization test (CPE-NT) from reindeer sera

Of the 55 borderline reindeer sera samples from IIFA, 42 (76%) of the samples were confirmed to be negative and had a titre ˂ 20 while 13 (24%) of the remaining samples were positive on neutralization test.

### Plaque reduction neutralization test (PRNT)

Sixty samples of the 480 reindeer sera with strongly positive (25), borderline (25), or negative (10) on IIFA were analyzed by PRNT. Of these 48 (80%) were confirmed seropositive and had neutralizing antibodies against INKV with titre ≥ 20, six samples (10%) were seronegative with titre ≤ 10 and six samples (10%) had no neutralization. Among the seropositives 39% (19/48) had titre ≥ 20–40, 52% (25/48) had titre ≥ 80–160, 8% (4/48) had titre ≥ 320. None of the samples showed any cross-reactivity with TAHV and SSHV.

## Discussion

The frequent movement of humans and animals across countries might contribute to the spread of viruses and vectors. Currently, there is no INKV surveillance in Norway. As part of the surveillance analysis of viral infections that may cause encephalitis, an IgG INKV IIFA was performed. We analysed reindeer sera as sentinel animals for the surveillance of possible circulation of INKV-infection in Norway. IIFA can be used in INKV monitoring programs to test antibodies in reindeer as well as for diagnosis of INKV infection in other animal species and humans.

Reindeer have previously been suggested to serve as amplification hosts for INKV [6]. The virus is mostly transmitted by *Ae. communis* mosquitoes feeding on large mammals, such as cattle, reindeer, and moose which show high INKV antibody prevalence [6]. Detection of INKV in mosquitoes and anti-INKV specific antibodies in reindeer are good indications that infected mosquitoes are circulating in the area posing a risk of transmission of INKV to humans in Norway. Interestingly, in our study, 62% of the reindeer investigated showed specific antibodies against INKV. This correlates well with previous studies on INKV seropositive from Finland in adult moose, reindeer and sheep with 64%, 89% and 7–75%, respectively [7, 45, 46]. In our study there was highest seroprevalence among the adult females and calves (Table 3). The seroprevalence of INKV among the calves (< 1 year of age) indicate recent exposure to the virus from the environment or by active immunization via mothers' milk [47].

IIFA for INKV is known to cross-react with other California serogroups of viruses such as TAHV and SSHV in Finland [33, 46]. Nevertheless, none of the reindeer sera analysed with the INKV neutralization test in this study cross-reacted with TAHV or SSHV. As IgG antibodies can be detected for several months, and up to years, or even a lifetime, after exposure to the virus [33] detection of INKV IgG among reindeer may reflect an old infection. However, it is likely that the seropositive reindeer found in Røros and Fosen may have acquired INKV the previous summer as the detection of the virus in the BSI confirmed the circulation of INKV in these regions. The lack of detection of INKV in the BSI pools at the other sites may be due to low viral load. Another possibility is lacking or low numbers of the actual vector species in the analysed BSI pool. It cannot be ruled out that the antibodies detected in reindeer sera could have been acquired from infections gained outside Norway since some reindeer cross the borders to Sweden, Finland, or Russia [48]. However, the cross-border herding is rather limited most places, and reindeer can be regarded as good sentinels for the local ecosystems, feeding blood sucking insects through the summer season. To get information about acute infections in reindeer, a test to detect IgM antibodies in their sera could have been performed. However, this was not possible due to lack of conjugates and positive controls. It is therefore essential to study IgM antibodies to INKV as a sign of recent infection among the human and animal population during mosquito seasons. High seropositivity of INKV in human populations has been indicated by recent studies in Sweden and Finland [11, 32]. A study in Norway demonstrated mean IgG prevalence against CE viruses of 20% among the militaries without any clinical symptoms [16]. It could be interesting to study if the exposure of reindeer to INKV have any impact on reindeer health in future studies.

The distribution and prevalence of INKV in mosquitoes in Norway is largely unknown. However, INKV is circulating in Northern Europe including Norway [2, 16]. Similarly, other viruses belonging to the CE group have been isolated from five different *Aedes* spp. in Norway, but the methods were not specific enough to identify and confirm the findings as INKV or if they represented other related CE group viruses. Those viruses were isolated from mosquitoes collected from Trandum and Øyern (Akershus), and Masi (Troms and Finnmark) in 1975 and Sjusjøen (Innlandet), Trysil (Innlandet) and Trandum (Akershus) in 1976 [7, 16].

In the present study, INKV were detected in two out of 213 pools of BSI collected in Fosen and Røros in the summer of 2014–2015, supporting previous findings indicating that INKV may be circulating in Norway [7, 16]. Although our results show that INKV was present at these two sites in 2014–2015, this does not signify that the virus was not present in these areas before 2014–2015, since these locations were not investigated previously. The low prevalence of the virus from these two regions may indicate that the virus might have been carried to these areas by animals returning from areas with higher prevalence of the virus. The overall EPP of INKV in Norway was calculated to be 0.04% [42]. With this low prevalence it is unlikely that a single pool contains more than one infected insect, however we cannot exclude the possibility of several, which would affect the calculation of the true virus prevalence in the population of BSI [49]. For true knowledge on the virus prevalence in an area, repeated samplings of mosquitoes over several years are necessary, or a confirmation of circulation of the virus in human and animal sera within the same location. The samples containing virus were relatively few in this study. By visual inspection and barcoding of these two pools we suggest that *Aedes* spp. are the biological vector of INKV in Norway, since INKV has been detected previously in adult *Aedes (Ae.) communis*, *Ae. hexodontus* and *Ae. punctor* mosquitoes and in *Ae. communis* larvae [7, 15–17]. The prevalence of INKV in the BSI population can possibly be affected by transovarial transmission of the virus, from infected mothers to the BSI progeny [1].

Many mosquitoes collected in this study were identified as *Aedes* spp. with lower numbers of *Culex* spp. SINV has mostly been isolated from *Culex* mosquitoes in studies from Sweden, and *Cx. torrentium* has been identified as the main vector of SINV in Scandinavia [22]. Nevertheless, the SINV/SINV-like virus have previously been isolated from *Aedes* in Norway [28, 35]. The first SINV strain isolated from mosquitoes in Norway (Vemork 1/92) was collected in 1992 in Rjukan (Vestfold and Telemark), Norway [35, 36]. The virus is found to be geographically limited in Norway with most of the cases of human SINV-infections being reported from Rjukan [26], which is situated south of the southernmost collection sites, Valdres, in our study. Since there were low numbers of *Culex.* spp*.* in the present study we cannot rule out the presence of SINV in the studied areas. Also, the presence of migratory birds could affect incidence of SINV. However, SINV could not be detected from the collected BSI pools by RT-real-time PCR in the present study. Increasing the sample size of BSI or pure mosquito pools might have increased the possibility to detect positives.

The findings in this study are important for public awareness about geographical distribution of the INKV. This knowledge may help to take necessary preventive measures against INKV infections in the future. In further studies it would be interesting to screen mosquitoes and the human population to determine the seroprevalence of INKV during the mosquito season, as well as whole genome sequencing of isolated INKV to identify which strains are circulating in Norway.

## Conclusion

There are knowledge gaps regarding the occurrence, distribution, and prevalence of the mosquito-borne viruses, INKV and SINV in Norway. The last study on these viruses was in 1992, reporting circulation of these viruses in the country. Our study is important to establish a baseline and provide new knowledge on the prevalence and distribution of INKV in BSI and reindeer. Moreover, it is important to monitor these viruses in case of an outbreak, and to avoid undiagnosed and unreported human cases. This study indicates that there is a low prevalence of INKV in BSI in Norway. The high seroprevalence of INKV in reindeer may indicate a risk of virus transmission from BSI to humans in these regions. Reindeer are one of the major hosts for INKV-infections and serve as an important sentinel animal for circulation of INKV. The occurrence and prevalence of INKV in vectors, animals, and the human population in Norway and other countries in Northern Europe requires further studies and monitoring.

## Supplementary Information

**Additional file 1: Table S1.** Distribution of IgG antibodies against INKV reindeer sera determined by IIFA for the winter seasons of 2013–2014, 2014–2015 and 2015–2016 with number of total positive/number total tested, seropositivity rate %, standard deviation (SD) and confidence interval (95% CI).

## Data Availability

The datasets used and/or analysed during the current study available from the corresponding author on reasonable request.

## Abbreviations

INKV: Inkoo virus
SINV: Sindbis virus
TAHV: Tahyna virus
BATV: Batai virus
SSHV: Snowshoe hare virus
CE: California encephalitis
RT-PCR: Reverse transcriptase PCR
EPP: Estimated pool prevalence
IIFA: Indirect immunofluorescence assay
CPE-NT: Cytopathic effect neutralization test
PRNT: Plaque reduction neutralization test
