# Supplementary material for: Inkoo and Sindbis viruses in blood sucking insects, and a serological study for Inkoo virus in semi-domesticated Eurasian tundra reindeer in Norway
Source: Virol J. 2022 Jun 3;19:99. doi: 10.1186/s12985-022-01815-0 (PMC9166600; doi:10.1186/s12985-022-01815-0)
Supplement: Supplementary file 1 — Additional file 1: Table S1. Distribution of IgG antibodies against INKV reindeer sera determined by IIFA for the winter seasons of 2013–2014, 2014–2015 and 2015–2016 with number of total positive/number total tested, seropositivity rate %, standard deviation (SD) and confidence interval (95% CI). [file 12985_2022_1815_MOESM1_ESM.pdf]

| Locations | Number of total positive/number total tested | Seropositivity rate % (SD) | 95% Confidence interval (CI) |
|---|---|---|---|
| **All locations** | | | |
| **2013-14** | 109/180 | 61 | |
| **2014-15** | 102/160 | 64 | |
| **2015-16** | 85/140 | 61 | |
| **Total period** | 296/480 | 62* (2.1) | 59.6-64.4 |
| **Tana** | | | |
| **2013-14** | 12/20 | 60 | |
| **2014-15** | 6/20 | 30 | |
| **2015-16** | 6/20 | 30 | |
| **2013-16** | 24/60 | 40* (17.3) | 20.4-59.6 |
| **Lakselv** | | | |
| **2013-14** | 10/20 | 50 | |
| **2014-15** | 13/20 | 65 | |
| **2015-16** | 17/20 | 85 | |
| **2013-16** | 40/60 | 67* (17.6) | 46.8-86.5 |
| **Tromsø** | | | |
| **2013- 14** | 13/21 | 62 | |
| **2014- 15** | 12/20 | 60 | |
| **2015-16** | 16/20 | 80 | |
| **2013-16** | 41/61 | 67* (11.0) | 54.9-79.8 |
| **Lødingen** | | | |
| **2013-14** | 12/22 | 55 | |
| **2014-15** | 15/20 | 75 | |
| **2015-16** | Nd | Nd | |
| **2013-16** | 27/42 | 64* (14.1) | 45.4-84.6 |
| **Hattfjelldal** | | | |
| **2013-14** | 18/30 | 60 | |
| **2014-15** | 12/20 | 60 | |
| **2015-16** | 13/20 | 65 | |
| **2013-16** | 43/70 | 61* (2.9) | 58.4-64.9 |
| **Fosen** | | | |
| **2013-14** | 13/20 | 65 | |
| **2014-15** | 18/20 | 90 | |
| **2015-16** | 9/20 | 45 | |
| **2013-16** | 40/60 | 67* (22.6) | 41.2-92.2 |
| **Røros** | | | |
| **2013-14** | 16/22 | 73 | |
| **2014-15** | 13/20 | 65 | |
| **2015-16** | 12/20 | 60 | |
| **2013-16** | 41/62 | 66* (6.6) | 58.6-73.4 |
| **Valdres** | | | |
| **2013-14** | 15/25 | 60 | |
| **2014-15** | 13/20 | 65 | |
| **2015-16** | 12/20 | 60 | |

| | | | |
|---|---|---|---|
| **2013-16** | 40/65 | 62* (2.9) | 58.4-64.9 |
